# Metropolitan Hospital Sunday Fund

**Published:** 1909-12-25

**Authors:** 


### METROPOLITAN HOSPITAL SUNDAY FUND.

### ANNUAL MEETING AT THE MANSION HOUSE.

THE annual meeting of the constituents of the Metropolitan Hospital Sunday Fund was held at the Mansion House on December 17, the Lord Mayor (Sir John Knill) presiding.

The report of the Council for the year ending October 31 stated that the year's collection had resulted in a total of £72,650 8s. 5d. The collections in the various places of worship resulted in a sum of £39,143, being £1,096 less than in 1908. St. Paul's Cathedral headed the list with £4,326, Christ Church, Lancaster Gate, coming next with £1,001. The report also referred to the special legacies from the executors of the late Mr. George Herring, the late Mr. Herbert Lloyd, the late Mr. Edwin Gayford, and the late Mr. C. E. Layton, and donations from Mr. William Herring and "Delta." The deaths of the following members of the Council were regretfully recorded: The Revs. Dr. Rigg and Dr. Marks, Sir Thos. Smith, Sir Stephen Mackenzie, and Mr. Thomas Wakley. It was announced that a sum of £67,212 had been distributed to 164 hospitals and institutions, 59 dispensaries, and 30 nursing associations, and that grants of 7,545 surgical appliances had been made.

Sir J. C. Bell moved the adoption of the report, and expressed the indebtedness of those responsible for the Fund to the clergy and ministers of all denominations.

The Rev. W. Hardy Harwood, of Union Chapel, Islington, seconded, and referred to the gratifying fact that, although there had been some diminution in the amount collected, the number of congregations contributing had risen to 2,070, representing a high-water mark from that point of view. If some churches had fallen off in the support they were able to give, this did not mean any slackening of interest in the Fund, but was generally due to the fact that the churches, particularly in the in-town districts, had greater demands upon them arising out of the conditions of their own neighbourhoods. As a representative of a number of churches, he could say that they had absolute confidence in the Fund and in the general excellence and regularity of its administration.

The motion was supported by the Rev. L. S. Lewis, of St. Mark's, Whitechapel, although, he said, no grant had been made to the hospital on behalf of which he pleaded last year. A grant was offered and refused—mistakenly, he thought—because the board of management regarded as official a statement made by one of the speakers at the last meeting, and thought that by accepting the grant they would either be untrue to the trust of their hospital or be guilty of receiving money under false pretences.

The report was adopted.

Archdeacon Sinclair moved a resolution fixing June 12 as Hospital Sunday, 1910, and inviting the cordial co-operation of all ministers of religion within the metropolitan area. He thought that the Sunday named would be the date least likely to clash with the occasion of any other special effort. He was glad to say that, in spite of certain difficulties, the past year had been a good one. More united efforts were wanted, and he urged that the clergy should introduce the claims of the Fund among those of their parishioners who were not at church on the particular Sunday on which the collection was taken. It was by such work as this that Canon Fleming, Canon Ridgeway, and others raised their large sums. It was good to realise, concluded the Archdeacon, the harmony of our great religious bodies in the cause of the relief of the sick. The differences that existed amongst them would doubtless be accentuated during the next few months, but the object for which they were gathered there that day could suffer from no political vicissitude.

The Rev. P. Clementi-Smith, in seconding, said that they in the City well understood the difficulty of collecting for the Fund, and if all were left to the Sunday effort the response would be poor. But a canvass of the offices in the City parish was an excellent way of greatly augmenting the contributions.

The resolution was then carried.

Lord Cheylesmore briefly moved: "That the Council for the year 1909 be re-elected for 1910, with the addition of Sir George Wyatt Truscott, Bt., John H. Morgan, Esq., C.V.O., F.R.C.S., the Rev. Morris Joseph, and the Rev. D. J. Waller to fill vacancies."

This was seconded from the body of the hall and carried.

Sir Savile Crossley, in moving a vote of thanks to the Lord Mayor, referred to the harmony with which the King's Hospital Fund, the Hospital Sunday Fund, and the Hospital Saturday Fund worked together. The resolution was briefly seconded by Mr. Thos. Bryant, and was carried, the Lord Mayor expressing his pleasure at presiding.

A motion was down on the agenda in the name of the Rev. L. S. Lewis in the following terms: "That in Law III. of the constitution all the words after 'nine other members' be deleted, and the following words substituted, 'who shall be elected by the constituents of the Fund at the annual general meeting.'"

Mr. Lewis said that he would withdraw it if he could obtain an assurance from the Chairman of the meeting that the constituents of the Fund possessed the right reasonably to criticise the report.

The Lord Mayor said that the constituents had an undoubted right of criticism, and he did not think that right had ever been refused.

Mr. Lewis dissented from the latter remark, but withdrew his motion.

The proceedings then concluded.

